# Exercise-Induced Myokines in Obesity-Related Metabolic Disorders and Cardiovascular Protection: A Narrative Review

**DOI:** 10.3390/sports14050212

**Published:** 2026-05-21

**Authors:** Yuxuan Zhang, Yajun Qiu

**Affiliations:** Department of Sports Science, College of Education, Zhejiang University, Hangzhou 310058, China; 3230105333@zju.edu.cn

**Keywords:** obesity, myokine secretion, exercise prescription, atherosclerosis, metabolic syndrome, HIIT, insulin resistance

## Abstract

Obesity is a significant risk factor for metabolic diseases and atherosclerotic cardiovascular disease (ASCVD). Exercise exerts beneficial effects partly through myokines secreted by skeletal muscle. This narrative review summarizes current evidence on exercise-induced myokines in obesity. We searched PubMed, Scopus, and Google Scholar up to Jan 2026 using keywords “myokines”, “obesity”, “resistance training”, “aerobic exercise”, and “HIIT”. We focused on six myokines (IL-6, irisin, FGF21, myostatin, apelin, and Metrnl) that are consistently linked to metabolic and cardiovascular health. Key findings are as follows: resistance training effectively increases irisin and decreases myostatin, promoting muscle mass and fat browning; high-intensity interval training (HIIT) induces rapid IL-6 peaks and elevates Metrnl, enhancing anti-inflammatory responses and cardiac function; aerobic exercise improves FGF21 sensitivity and supports long-term metabolic homeostasis. For clinicians and exercise practitioners, a preliminary exercise framework can be suggested based on available human evidence. In obese patients, ≥3 sessions per week of resistance training (60–80% of one-repetition maximum, 8–12 repetitions, 3–4 sets) may be considered to optimize irisin/myostatin balance, combined with ≥150 min per week of moderate-intensity aerobic exercise (50–70% of maximum heart rate) or 75 min per week of HIIT (85–95% of peak heart rate, 4 × 4 min intervals) to improve FGF21 sensitivity and Metrnl levels. These suggestions should be interpreted as hypothesis-generating rather than definitive clinical guidance, given the heterogeneity of included studies and the absence of quantitative synthesis. Nevertheless, they offer a molecular basis for hypothesis-driven precision exercise prescription that requires validation in future prospective studies and randomized controlled trials.

## 1. Introduction

Obesity has become a global public health challenge. As a core component of metabolic syndrome, it is not only a significant contributor to metabolic diseases such as type 2 diabetes and non-alcoholic fatty liver disease but also one of the most critical and modifiable risk factors for atherosclerotic cardiovascular disease (ASCVD). In the state of obesity, adipose tissue dysfunction leads to increased release of free fatty acids, elevated secretion of pro-inflammatory adipokines, and reduced secretion of anti-inflammatory adipokines, thereby triggering chronic low-grade inflammation and insulin resistance. These metabolic disturbances further impair vascular endothelial function, promote oxidative stress, lipid deposition, and vascular wall inflammation, ultimately driving the formation and progression of atherosclerotic plaques. Obesity is thus regarded as the “soil” for cardiovascular diseases, and its associated metabolic abnormalities serve as a key bridge linking obesity to cardiovascular events [1,2].

Against this backdrop, exercise, as a non-pharmacological intervention, has been proven to effectively ameliorate obesity-related metabolic abnormalities and reduce cardiovascular risk [3,4,5]. However, its precise mechanisms of action are not yet fully elucidated. In recent years, skeletal muscle has been re-recognized as an active endocrine organ capable of synthesizing and releasing a series of bioactive molecules in response to exercise stimulation, known as “myokines”. The discovery of myokines stemmed from the work of Pedersen et al. in 2003, who first demonstrated that skeletal muscle could synthesize and release large amounts of interleukin-6 (IL-6) into the circulation during exercise, thereby regulating the metabolism and function of distant organs [6]. This seminal finding marked the birth of the first clearly defined myokine and established skeletal muscle as an endocrine organ beyond its classical role in locomotion. Since then, with advances in proteomics and metabolomics, hundreds of myokines have been identified [7,8,9]. These factors act as molecular messengers between exercise and other organs, including adipose tissue, liver, and vasculature. To date, hundreds of myokines have been identified, among which irisin, FGF21, myostatin (MSTN), apelin, and Metrnl have been demonstrated to have direct associations with obesity-related metabolic abnormalities and cardiovascular protection [7,9]. Different exercise modalities, including resistance training, aerobic exercise, and high-intensity interval training (HIIT), can induce characteristic myokine secretion profiles [10,11], which in turn influence metabolic and cardiovascular outcomes through distinct signaling pathways. Systematically elaborating on the secretion characteristics of these myokines and their roles in obesity-related metabolic disorders and cardiovascular protection is of great significance for understanding the precise mechanisms of exercise intervention and optimizing exercise prescriptions. This review summarized the biological characteristics of major exercise-induced myokines, the regulatory patterns of their secretion by different exercise modalities, and their mechanisms of action in improving obesity-related metabolic abnormalities and cardiovascular health (Table 1). It seeks to provide a theoretical basis and molecular perspective for the application of exercise in the prevention and treatment of metabolic diseases and cardiovascular protection.

## 2. Method

This narrative review was conducted following the SANRA (Scale for the Assessment of Narrative Review Articles) guidelines to ensure transparency. Given the narrative nature of the review, a systematic meta-analysis was not performed; however, explicit criteria were applied to literature selection.

### 2.1. Literature Search Strategy

A systematic search of PubMed, Scopus, and Google Scholar was performed from database inception to January 2026. The search terms combined keywords related to myokines (“myokine”, “irisin”, “FGF21”, “myostatin”, “MSTN”, “apelin”, “Metrnl”, “IL-6”), obesity (“obesity”, “overweight”, “adiposity”), and exercise (“exercise”, “resistance training”, “aerobic exercise”, “high-intensity interval training”, “HIIT”). Boolean operators (AND, OR) were used to combine terms. The full search strings for each database are provided in Supplementary File S1.

### 2.2. Inclusion and Exclusion Criteria

Included studies were: (1) original research articles (randomized controlled trials, prospective cohorts, cross-sectional studies, and human intervention studies); (2) involving overweight or obese participants (BMI ≥ 25 kg/m^2^ or clinical diagnosis of obesity); (3) containing an exercise intervention (acute or chronic), comparing exercise modalities, or being an observational study that measured myokine levels in relation to obesity or exercise status; (4) measuring at least one myokine in blood, muscle, or other tissues; and (5) published in English. Exclusion criteria were: conference abstracts, case reports, and studies without original data. Systematic reviews and meta-analyses were not used as primary evidence but are cited for background context. Animal studies were not used as primary evidence and were only mentioned in mechanistic discussions with explicit labeling.

### 2.3. Selection of Myokines

Among the hundreds of identified myokines, we focused on six—irisin, FGF21, myostatin (MSTN), apelin, Metrnl, and IL-6—based on the following criteria: (a) established or strongly suggested direct links to obesity-related metabolic dysregulation; (b) consistent evidence of exercise-induced regulation in human studies; (c) plausible mechanistic connections to cardiovascular protection; and (d) sufficient number of published human studies (as summarized in Supplementary Table S1) to allow meaningful interpretation. Other myokines (e.g., IL-15, BDNF) and mitochondrial-derived peptides (MOTS-c, Humanin) are briefly mentioned but not discussed in depth due to limited human data specific to obesity.

A total of 34 original human studies focusing on overweight or obese participants (BMI ≥ 25 kg/m^2^ or clinical obesity) met the inclusion criteria; their characteristics are summarized in Supplementary Table S1. The majority were randomized controlled trials and prospective cohorts, complemented by cross-sectional studies and human intervention or cellular investigations. A narrative synthesis was performed: for each myokine, we summarized the direction and magnitude of exercise-induced changes reported across studies, noted the consistency or inconsistency of findings, and categorized the results according to exercise modality (resistance, aerobic, HIIT) and timing of measurement (acute vs. chronic). Where multiple studies reported contradictory results, we integrated them using the resistance-adaptation model rather than simply listing opposing findings.

### 2.4. Data Extraction and Synthesis

From each included study, we extracted: first author, year, study design, participant characteristics (sample size, age, sex, BMI), exercise modality (type, intensity, duration, frequency), myokine measured (assay method, timing of measurement), and main findings. A summary of the extracted data is provided in Supplementary Table S1. Due to heterogeneity in study designs and outcome measurements, a quantitative meta-analysis was not feasible; instead, findings were synthesized narratively, with emphasis on consistent patterns across studies and transparent reporting of contradictory results. The quality of this narrative review was assessed using the SANRA tool. As shown in Supplementary Table S2, the review meets five of the six SANRA criteria fully; item 3 (description of literature search) is partially met due to the narrative design, but the complete search strings are provided in Supplementary Methods. Findings were organized thematically according to the six selected myokines, with additional subsections for other myokines and methodological considerations.

## 3. Myokine Secretion Profiles Induced by Different Exercise Modalities

### 3.1. IL-6

Interleukin-6 (IL-6) is a pleiotropic cytokine with context-dependent functions. Under resting conditions, particularly in obese and sedentary populations, elevated circulating IL-6 is primarily derived from adipose tissue macrophages and reflects chronic low-grade inflammation via NF-κB pathway activation, associating with insulin resistance and metabolic abnormalities [29]. However, during exercise, contracting skeletal muscle becomes the predominant source of IL-6, with its expression driven by intracellular calcium flux and energy status through JNK and AP-1 signaling pathways [30] (in vitro human cell study). This distinction is crucial: while adipose-derived IL-6 serves as a marker of pathological inflammation, muscle-derived IL-6 functions as a beneficial metabolic regulator.

Exercise-induced IL-6 release exhibits characteristic kinetics: circulating levels increase exponentially during exercise, rising up to 100-fold above resting concentrations, with peak levels occurring immediately after concentric exercise or approximately 8 h after eccentric exercise, followed by rapid decline to baseline [15,31]. Different exercise modalities produce distinct IL-6 secretion patterns. High-intensity interval training (HIIT), due to its intense metabolic stress, induces an extremely rapid and sharp peak in IL-6 release [12,13]. Prolonged moderate-intensity aerobic exercise leads to a more sustained and cumulative secretion pattern, with levels remaining elevated throughout the exercise bout [31]. Resistance training also increases circulating IL-6, though some studies suggest that IL-6 responses to resistance training may be less sensitive to intensity variations [12,13]. Exercise duration and volume are key determinants, with prolonged duration or increased volume typically leading to higher peak levels [31]. While most studies consistently report acute exercise-induced IL-6 elevation, comparisons between modalities remain inconclusive. Some studies demonstrate that HIIT produces higher peak IL-6 levels than traditional aerobic exercise [12], while others report comparable responses when total work is matched. These discrepancies may arise from differences in exercise protocols (intensity, duration, recovery intervals), participant characteristics (training status, baseline inflammation), and sampling timing given IL-6 rapid kinetics.

Acutely elevated muscle-derived IL-6 plays a central role in energy homeostasis during exercise. It acts directly on the liver to promote gluconeogenesis, maintaining blood glucose stability, while simultaneously stimulating adipose tissue lipolysis to increase free fatty acid mobilization and oxidation for contracting muscles [32,33]. Importantly, this pulsatile IL-6 signal induces expression of anti-inflammatory cytokines such as IL-10 and IL-1 receptor antagonist (IL-1Ra) in skeletal muscle and adipose tissue, initiating a systemic acute anti-inflammatory response [34,35]. In cardiovascular health, IL-6 exhibits duality depending on downstream signaling pathways. Under pathological conditions like atherosclerosis, the “trans-signaling” pathway mediated by soluble IL-6 receptors predominates, promoting vascular inflammation, monocyte recruitment, and plaque instability [14,36]. In contrast, exercise-induced muscle-derived IL-6 is thought to preferentially activate the “classical signaling” pathway via membrane-bound receptors based on indirect evidence from human studies and mechanistic data from cell models; this pathway is associated with anti-inflammatory and cardioprotective effects [34,35]. Regular exercise thus remodels IL-6 signaling balance—enhancing protective classical signaling while indirectly attenuating harmful trans-signaling through improved inflammatory status [37]. Taken together, while the magnitude of IL-6 elevation varies by exercise modality, the overall evidence supports an acute, activity-dependent release of muscle-derived IL-6 that mediates beneficial metabolic and anti-inflammatory effects, with HIIT offering the most robust response.

### 3.2. Irisin

Irisin is a myokine that promotes white adipose tissue browning, originally identified through animal studies [38] and subsequently confirmed in humans [39]. Its synthesis is directly coupled to exercise via PGC1α upregulation during muscle contraction, which enhances FNDC5 expression followed by proteolytic cleavage and release of irisin into circulation [39]. In animal models and human cell studies, irisin has been shown to act on white adipose tissue to induce “browning”—increasing expression of thermogenic proteins such as UCP1—thereby promoting energy expenditure [39]. Direct evidence in human adipose tissue in vivo remains limited. It also exerts effects on bone, brain, and cardiovascular tissues. Different exercise modalities differentially regulate irisin secretion. Regarding exercise type, a randomized crossover trial demonstrated that resistance training significantly increased circulating irisin in healthy men, whereas endurance training alone did not [16]. A meta-analysis confirmed that resistance training is more effective than aerobic training for elevating circulating irisin [17]. Studies in elderly populations further suggest that higher-intensity resistance training produces more pronounced effects [40]. Regarding exercise intensity, under conditions of equivalent energy expenditure, high-intensity exercise induces greater serum irisin increases compared to low-intensity exercise, indicating a positive correlation between post-exercise irisin levels and exercise intensity [18,19]. This positions HIIT as a potentially effective strategy for rapidly elevating irisin. Environmental temperature also influences irisin responses, though findings on long-term training remain controversial. It has been reported that endurance training increases circulating irisin in healthy adults [41]. However, some studies report decreased circulating irisin in obese populations following training, potentially related to improvement of irisin resistance—a compensatory elevation in obesity due to reduced target tissue sensitivity [19,42,43]. To help interpret these discrepancies, we propose a tentative conceptual framework—“resistance-adaptation model”—as a hypothesis to be tested in future studies. It should be emphasized that this model remains speculative and currently lacks direct experimental validation in humans. In obesity, chronic low-grade inflammation may induce a state of irisin resistance: baseline circulating irisin is often elevated as a compensatory response, but target tissue sensitivity is reduced [42,43]. Exercise training could act through two phases. Acutely, each bout triggers an intensity-dependent irisin peak; this peak may be blunted in individuals with severe insulin resistance. Chronically, regular training (especially resistance training and HIIT) reduces systemic inflammation and restores tissue sensitivity, which lowers the compensatory high baseline irisin toward normal levels. This would explain why some long-term studies report decreased irisin after training [19]—not a paradox, but a sign of resolved resistance. Conversely, studies measuring irisin immediately after an acute exercise bout (rather than at resting baseline) tend to report increases. Therefore, contradictory findings arise largely from differences in metabolic status (obese vs. lean), sampling timing (post-acute vs. resting chronic), and training duration. The resistance-adaptation model is presented here as an interpretive tool, not as a proven physiological mechanism. Direct experimental testing is required to confirm or refine this framework.

Metabolically, irisin is recognized for its ability to promote energy expenditure and induce browning of white adipose tissue, thereby contributing to metabolic benefits [39]. Irisin levels negatively correlate with obesity indicators including BMI and fat mass [44,45]. Irisin also activates mTOR and ERK signaling pathways to promote muscle protein synthesis and satellite cell activation [46,47], suggesting dual benefits for improving body composition. Regarding cardiovascular effects, clinical evidence shows negative correlations between irisin levels and coronary artery disease severity, with higher irisin associated with improved prognosis [48]. Notably, some studies report positive correlations between irisin and carotid intima-media thickness [49], possibly reflecting compensatory secretion during disease progression or target organ resistance—again emphasizing context-dependent interpretation. Taken together, resistance training and HIIT consistently elevate circulating irisin, whereas the effect of aerobic training is less uniform; the discrepancies are largely reconciled by the resistance-adaptation model accounting for metabolic status and sampling timing.

### 3.3. FGF21

Fibroblast growth factor 21 (FGF21) is a polypeptide hormone belonging to the fibroblast growth factor family, recognized for its multifaceted roles in regulating glucose and lipid homeostasis and energy metabolism [50]. Unlike classical FGFs, FGF21 functions as an endocrine factor with pleiotropic metabolic effects. It is expressed in multiple organs including liver, white and brown adipose tissue, skeletal muscle, pancreas, heart, and brain, and can be induced under stress conditions such as exercise, cold exposure, and nutritional imbalance. While the liver is the primary source of circulating FGF21 under most conditions, skeletal muscle has been shown to secrete FGF21 during exercise, particularly under metabolic stress [50]. This multi-organ source underscores the role of FGF21 as an integrated stress-responsive hormone.

The secretion of FGF21 under exercise stress exhibits characteristic kinetic patterns. After acute exercise, circulating FGF21 levels rise rapidly, typically peaking around 1 h post-exercise and returning to baseline approximately 3 h later [50]. This rapid response suggests FGF21 functions as an early mediator of exercise-induced metabolic adaptation. Both aerobic and resistance exercise effectively modulate the FGF21 system, though with potentially different characteristics. A randomized controlled trial in obese men with type 2 diabetes demonstrated that both aerobic and resistance exercise improved FGF21 levels, with resistance exercise potentially showing a more substantial adaptive response [20]. The underlying mechanism may involve resistance exercise more effectively inducing FGF21 activation, thereby enhancing insulin action and improving AMPK activity in muscle.

The variability in FGF21 responses to exercise could be tentatively interpreted using the resistance-adaptation framework introduced earlier (Section 3.2), though this framework remains to be validated. In obesity and type 2 diabetes, FGF21 resistance is common: circulating levels are elevated but receptor signaling is impaired, as documented in human studies [51,52,53]. Exercise training restores tissue sensitivity [20], which may lower the compensatory high baseline FGF21—an effect that would be observed only when measuring resting levels after chronic training. In contrast, acute exercise transiently increases FGF21 in a duration- and intensity-dependent manner. Therefore, studies that measure FGF21 immediately after a single bout (particularly prolonged or high-intensity exercise) report elevation, whereas those measuring resting levels after weeks of training may report no change or even a decrease. By separating acute from chronic measurements and accounting for baseline resistance status, the seemingly contradictory findings become consistent.

Metabolically, FGF21 confers core benefits by repairing mitochondrial function, improving insulin resistance, and promoting white adipose tissue browning [54]. Through reconstructing effective FGF21 signal transduction, exercise enables FGF21 to combat the pathological basis of obesity, diabetes, and fatty liver. The modality-specific effects may reflect different emphases: resistance training appears more effective in activating FGF21-related pathways within muscle and enhancing insulin action locally, while endurance training may focus more on coordinating systemic glucose and lipid metabolism balance between liver and adipose tissue via FGF21 [20].

Cardiovascularly, FGF21 serves as an important signaling molecule mediating exercise-induced cardioprotection. At the vascular level, FGF21 exhibits anti-atherosclerotic potential, such as increasing adiponectin levels and activating the ACE2/angiotensin-(1-7) axis [54]. Differences in exercise-induced FGF21 secretion profiles may influence the emphasis on activating these protective mechanisms. Resistance training, by effectively improving body composition and basal metabolic rate, may primarily serve cardiovascular health through strengthening the indirect metabolic benefits of FGF21. In contrast, aerobic or high-intensity training may rely more on periodic, pulsatile FGF21 elevations to directly maintain vascular endothelial function. The restoration of FGF21 sensitivity by regular exercise—overcoming resistance states—represents a fundamental mechanism through which exercise re-establishes this hormone’s protective signaling.

Overall, both resistance and aerobic exercise improve FGF21 sensitivity, with resistance training potentially inducing a stronger adaptive response; acute exercise transiently elevates FGF21, while chronic training lowers compensatory high baseline levels in obese individuals.

### 3.4. MSTN

Myostatin (MSTN), also known as growth differentiation factor 8 (GDF-8), is a member of the transforming growth factor-beta (TGF-β) superfamily. It is primarily expressed in skeletal muscle and functions as a key negative regulator of muscle growth and development [55]. MSTN exerts its effects by binding to activin receptor type IIB (ActRIIB), leading to phosphorylation of SMAD2/3 and subsequent inhibition of myogenic gene transcription [55]. Beyond its canonical role in muscle, MSTN is also expressed in adipose tissue and has been implicated in metabolic regulation and cardiovascular pathology. In obesity and related metabolic abnormalities, MSTN plays a detrimental role. Clinical observations consistently demonstrate that circulating MSTN concentrations are elevated in obese populations and negatively correlate with insulin sensitivity [56]. These findings indicate that abnormally elevated MSTN levels exacerbate metabolic disorders through multiple mechanisms: inhibiting muscle growth, promoting fat accumulation, and impairing insulin signaling.

Exercise downregulates MSTN expression, with the magnitude of reduction depending on exercise mode, duration, and intensity. Mechanistic insights from cell and animal studies suggest that when muscle is subjected to mechanical stress, it can downregulate MSTN expression via pathways such as JNK-mediated phosphorylation of transcription factor Smad2 [55,57]. Acute exercise significantly reduces skeletal muscle MSTN levels, and the extent of downregulation is positively correlated with exercise duration [58,59]. Long-term regular exercise persistently lowers baseline MSTN levels in both skeletal muscle and circulation, and performing acute exercise again after long-term training leads to further reduction in MSTN expression [21,22,59]. Resistance training is the most effective exercise modality for downregulating MSTN expression. Studies indicate that both former weightlifters and sedentary men show decreased MSTN mRNA levels in leg muscles after acute resistance training [21]. The molecular mechanisms underlying resistance training-induced MSTN suppression involve multiple signaling pathways, including inhibition of the classical SMAD pathway and modulation of non-SMAD pathways such as MAPK/ERK, p38, JNK, and Akt phosphorylation [55]. The regulatory effect of aerobic endurance exercise on MSTN remains unclear and controversial.

Metabolically, exercise-induced MSTN downregulation represents an important mechanism through which exercise enhances muscle mass, improves body composition, and increases insulin sensitivity [21,22,59]. Resistance training, through its characteristic mechanical load, efficiently activates JNK/Smad pathways to inhibit MSTN expression [55]. This promotes increased lean body mass. As the primary organ for glucose disposal, increased muscle mass fundamentally expands metabolic capacity and increases basal energy expenditure, thereby counteracting obesity-related metabolic disturbances. The negative correlation between MSTN levels and insulin sensitivity [56] further supports the role of MSTN as a mediator of exercise-induced metabolic improvements.

In cardiovascular health, particularly atherosclerosis, MSTN exhibits direct pathological promoting effects. MSTN expression is extremely low in normal vascular tissue but significantly enriched in atherosclerotic lesions, with expression levels positively correlating with the severity of vascular injury. At the mechanistic level, MSTN exerts multiple damaging effects on endothelial function. For example, by activating TGF-β signaling, MSTN inhibits endothelial nitric oxide synthase (eNOS) phosphorylation, reducing NO production and leading to impaired vasodilation; it also upregulates vascular cell adhesion molecule-1 (VCAM-1) expression, promoting monocyte adhesion to the endothelium and initiating vascular wall inflammation [60]. Additionally, MSTN upregulates monocyte chemoattractant protein-1 (MCP-1), forming a positive feedback loop of inflammation that accelerates inflammatory cell infiltration and lipid deposition [60]. The clinical relevance of MSTN in cardiovascular disease is underscored by observations that atherosclerosis patients often suffer from sarcopenia, and MSTN levels independently correlate with low skeletal muscle mass, suggesting the existence of a “muscle-vascular axis” where MSTN may act as a common mediator linking skeletal muscle metabolic disorders and vascular pathology [61]. Therefore, long-term regular exercise, especially resistance training modalities that continuously lower circulating and tissue MSTN levels, produces cardiovascular protective effects through multiple mechanisms: attenuating direct toxic effects of MSTN on the vascular system, improving systemic metabolic status, and preserving endothelial function. By effectively downregulating MSTN, exercise simultaneously improves metabolic status and protects the vasculature, positioning MSTN and its signaling pathway as potential therapeutic intervention targets. In summary, resistance training robustly downregulates myostatin expression in a dose-dependent manner, whereas aerobic exercise has minimal effect; this suppression contributes to improved muscle mass and metabolic health.

### 3.5. Apelin

Apelin, a myokine synthesized and released by skeletal muscle during exercise, functions through the activation of G protein-coupled receptors. Endurance training has been shown to markedly upregulate its expression [23]. Following post-translational processing of its precursor, Apelin yields several bioactive fragments, with APLN-13 exhibiting the highest potency [62]. The Apelin-APJ receptor pair forms a signaling axis highly responsive to physical activity.

The impact of various exercise modalities on Apelin secretion presents a complex and often inconsistent picture. The apparent inconsistency in apelin responses to exercise could also be considered in light of the resistance-adaptation model (Section 3.2), but this interpretation requires direct experimental confirmation. In obese individuals with metabolic dysfunction, baseline apelin is often elevated as a compensatory mechanism. Aerobic exercise training may lower this elevated baseline by improving insulin sensitivity and reducing adipose inflammation—leading to an overall decrease or no net change depending on timing of measurement. In contrast, acute exercise (especially HIIT) can trigger a transient rise in apelin, which may be more pronounced in metabolically healthy individuals. This explains why some studies report significant elevation while others find no change—the former often measure apelin acutely post-exercise, the latter at resting baseline after chronic training. Similarly, whether HIIT appears superior to moderate-intensity continuous training depends on the timing of sampling relative to the last exercise bout. When apelin is measured acutely after a HIIT session, higher peaks are observed; when measured at resting baseline after a training period, both modalities yield similar levels [24]. Furthermore, the Apelin response to exercise demonstrates considerable inter-individual variation, heavily influenced by factors like gender and metabolic health. Notably, exercise increases apelin expression in obese men but not in women, regardless of adiposity status [25].

In terms of cardiovascular protection, the Apelin system functions through multiple mechanisms. Clinical observations find that reduced circulating Apelin levels are associated with coronary artery disease, particularly more evident in patients with unstable angina, suggesting a role in plaque stabilization [26]. In vitro studies using human cells and tissues indicate that apelin-13 improves lipid metabolism [62], exerts anti-inflammatory effects by inhibiting IL-6, IL-1β, and TNF-α, and may help stabilize atherosclerotic plaques [62,63]. Additionally, the APJ receptor can antagonize angiotensin II-mediated transcriptional regulation, helping maintain stable blood pressure and inhibit pathological vascular remodeling. Notably, the cardioprotective effects of Apelin partly depend on endothelial cell signaling in skeletal muscle and adipose tissue, providing a precise molecular connection point for the “exercise-muscle-vascular” axis [64]. In summary, the resistance-adaptation model offers a potential explanation for the seemingly contradictory apelin responses to different exercise modalities, and the available human evidence supports apelin as a beneficial mediator of exercise-induced cardiovascular improvements.

### 3.6. Metrnl

Metrnl (also known as meteorin-like protein) is a myokine whose expression is upregulated in response to exercise, particularly high-intensity and resistance training. Metrnl was originally identified as a secreted protein induced by exercise and cold exposure in murine models [65]. Based on studies in rodent muscle, exercise-induced elevation of PGC-1α stimulates Metrnl mRNA transcription, and AMPK activation further enhances this effect, promoting Metrnl secretion.

The temporal pattern of Metrnl response differs markedly between acute and chronic exercise. A single exercise bout induces a rapid but transient increase in Metrnl mRNA within human skeletal muscle, observed within 1–4 h after HIIT or combined exercise sessions [27]. Notably, this swift transcriptional activation is not immediately mirrored in the circulation, as plasma Metrnl protein levels typically remain unchanged after acute exertion. This discrepancy suggests that a detectable systemic release may depend on more prolonged or recurrent stimuli. In contrast, sustained exercise training leads to stable elevations in both Metrnl expression and its circulating concentration. Long-term exercise training can also increase circulating Metrnl levels and muscle mRNA expression in human [27,28,66].

The efficacy of various exercise modalities in modulating Metrnl is not uniform. Interventions characterized by high intensity or resistance components generally provoke more robust responses. Circuit resistance training has been shown to boost plasma Metrnl in overweight and type 2 diabetic individuals [67,68]. HIIT appears particularly potent, significantly enhancing Metrnl mRNA expression acutely and over longer periods in healthy humans [27]. Collectively, evidence indicates that high-intensity and resistance-based training are effective strategies for elevating Metrnl, with some data suggesting HIIT may offer advantages over moderate-intensity continuous training.

The relationship between Metrnl and metabolic health is multifaceted, with circulating levels exhibiting variable patterns across different metabolic states. Research indicates that individuals with type 2 diabetes or obesity may present with either elevated [69,70] or reduced concentrations of this myokine [71]. Notably, lower levels are often linked to inferior glycemic control and adverse lipid profiles. In obese populations, Metrnl demonstrates inverse correlations with HDL-C and positive associations with LDL-C, triglycerides, and total cholesterol [71], highlighting its potential role in lipid homeostasis that appears sensitive to physiological context.

Clearer mechanistic insights emerge from exercise intervention studies. Specific training regimens—such as high-intensity interval training (HIIT), circuit resistance training, and interval resistance training—consistently elevate circulating Metrnl [27,28,67,68]. This increase is frequently paralleled by enhancements in key metabolic parameters, including fasting glucose, insulin resistance indices, body composition, and overall lipid profiles. Intriguingly, the rise in Metrnl can occur independently of insulin level changes, pointing to its involvement in alternative, non-insulin-dependent glucose uptake pathways that may contribute to the metabolic benefits of exercise [70].

Within the cardiovascular domain, Metrnl exhibits compelling protective associations. Clinically, its circulating levels are diminished in patients suffering acute myocardial infarction, where lower concentrations correlate with greater disease severity and heightened long-term risk [72]. Furthermore, Metrnl levels are implicated in the trajectory of atherosclerosis, serving as an independent prognostic marker for disease progression [72,73]. Exercise-mediated increases in Metrnl reinforce its protective character; in patients with coronary artery disease, training-induced augmentation of skeletal muscle-derived Metrnl coincides with mitigated atherosclerosis, characterized by improved endothelial metabolism and reduced pro-inflammatory activity [66]. Elevated post-exercise Metrnl is favorably associated with lower LDL-C and inflammatory markers, higher HDL-C, and reduced clinical severity of coronary disease. Thus, HIIT and resistance training effectively elevate Metrnl levels, with HIIT showing a more pronounced acute response; chronic training increases circulating Metrnl, which is associated with improved cardiometabolic outcomes.

### 3.7. Other Myokines and Related Molecules

Exercise can induce skeletal muscle to produce and release a large number of myokines, which reach target organs via the bloodstream and exert biological effects. Besides the myokines discussed above, numerous other exercise-induced myokines are closely related to obesity. For example, IL-15 can serve as a predictor for early atherosclerosis in obese patients with non-alcoholic fatty liver disease (NAFLD), playing an important role in metabolic regulation and inflammatory responses [74]. Additionally, factors like BDNF can be induced by exercise and released from skeletal muscle, contributing to metabolic regulation [75].

Beyond the myokines mentioned, there is a class of novel signaling molecules called “mitochondrial-derived peptides” (MDPs). These are peptides encoded by mitochondrial DNA, primarily distributed in tissues like skeletal muscle and blood, and also play important roles in regulating body energy metabolism. Currently identified MDPs include MOTS-c, Humanin, and SHLP1-6 [76]. In humans, exercise has been shown to increase the expression of Humanin and SHLP6, exerting anti-inflammatory, insulin-sensitizing, and metabolic regulatory effects [77,78].

### 3.8. Comparison of Exercise Modalities at the Cellular Level in Humans

**Figure 1:**
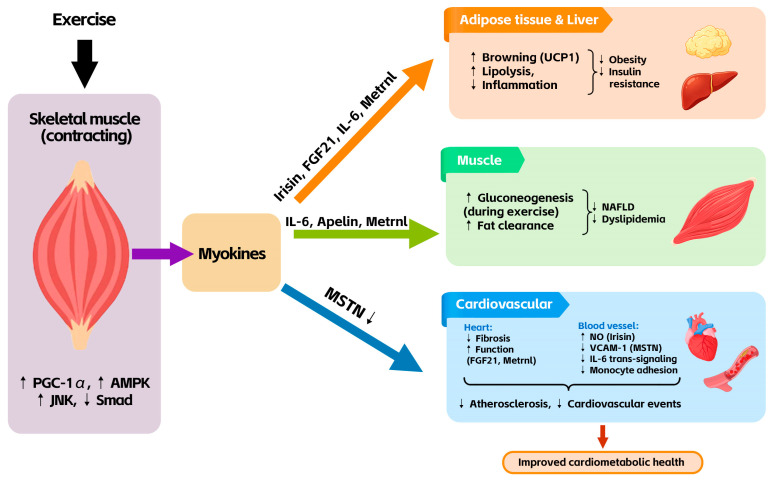
Schematic diagram of the myokine network in obesity. Exercise induces skeletal muscle to secrete multiple myokines (irisin, FGF21, IL-6, MSTN, apelin, Metrnl). These factors target adipose tissue, liver, blood vessels, and heart, collectively improving insulin sensitivity, promoting fat browning, reducing inflammation, and protecting against atherosclerosis.

The molecular crosstalk between muscle, adipose tissue, liver, and vessels is summarized in Figure 1. Note on mechanistic evidence: Most of the detailed signaling pathways described below have been primarily characterized in animal or cellular models; their direct extrapolation to human physiology requires caution. Where human data are available, they are cited; preclinical evidence is explicitly indicated.

Although Table 1 summarizes modality-specific myokine responses, the underlying cellular mechanisms that explain why one exercise mode may be superior to another require direct comparison. In human skeletal muscle, resistance training primarily activates mechanical stress-sensitive pathways. The tension generated during muscle contraction is sensed by integrins and focal adhesion kinase (FAK), leading to JNK phosphorylation. JNK directly phosphorylates Smad2/3 and inhibits their nuclear translocation, thereby downregulating MSTN expression [55]. Concurrently, mechanical load promotes protein synthesis via the mTORC1 pathway and upregulates PGC-1α-dependent irisin secretion [39]. Aerobic exercise predominantly triggers metabolic stress: an increased AMP/ATP ratio activates AMPK, and calcium release from the sarcoplasmic reticulum activates calmodulin-dependent kinases (CaMK). Both AMPK and CaMK phosphorylate PGC-1α, enhancing FGF21 and irisin expression [20,50], but without the strong MSTN suppression seen with resistance training. HIIT combines intense metabolic perturbation with intermittent recovery, producing dramatic energy fluctuations and hypoxic stimuli that elicit a rapid, high-amplitude IL-6 peak. This IL-6 signal acts through the classical receptor (not trans-signaling) to initiate transcription of anti-inflammatory cytokines (IL-10, IL-1Ra) [14]. In addition, HIIT strongly activates AMPK and PGC-1α, inducing Metrnl expression [27]. Thus, at the cellular level, resistance training uniquely suppresses MSTN via the JNK/Smad axis; aerobic exercise enhances FGF21 sensitivity through sustained metabolic stress; HIIT generates rapid anti-inflammatory and cardioprotective signals via pulsatile metabolic perturbation. The resistance training-associated JNK/Smad pathway (MSTN suppression) and the HIIT-associated classical IL-6 signaling have been largely derived from animal and cellular studies, as indicated in the references. Direct human evidence for these specific phosphorylation events remains limited. These distinct cellular mechanisms may help guide exercise prescription according to the predominant metabolic deficit. For example, resistance training could be particularly suitable for sarcopenic obesity, as it suppresses myostatin and promotes irisin-mediated fat browning; aerobic exercise might benefit insulin resistance by enhancing FGF21 sensitivity through sustained metabolic stress; and HIIT may be advantageous for individuals with high inflammatory risk, given its ability to induce a rapid anti-inflammatory IL-6 pulse and elevate Metrnl. 

## 4. Implications for Exercise Prescription and Therapeutic Strategies

The differential secretion profiles of exercise-induced myokines, shaped by distinct exercise modalities, have important implications for optimizing exercise interventions and developing novel therapeutic approaches for metabolic and cardiovascular diseases.

### 4.1. Modality-Specific Myokine Profiles Guide Exercise Prescription

The distinct myokine responses to different exercise modalities provide a molecular rationale for tailoring exercise prescriptions based on specific health goals. Resistance training appears to be the most effective strategy for downregulating MSTN [21]. This modality has also been reported to elevate irisin levels [16,17], suggesting it may be particularly suitable for individuals with sarcopenic obesity, as it simultaneously targets muscle hypertrophy and fat browning. The dual action of resistance training—promoting muscle protein synthesis via mTOR and ERK signaling while enhancing energy expenditure through irisin-mediated pathways—may offer potential advantages for improving body composition [46,47]. Based on the available human evidence, a preliminary exercise prescription framework is outlined in Table 2.

High-intensity interval training (HIIT) has been associated with rapid and pronounced peaks of IL-6 and robust induction of Metrnl, suggesting potential benefits for enhancing acute anti-inflammatory responses and improving myocardial metabolism [27,28,66]. The intense metabolic stress induced by HIIT triggers a pulsatile IL-6 release that activates anti-inflammatory cascades [14,34], while Metrnl upregulation has been associated with improved cardiac function in human studies [66]. These features suggest that HIIT could be a time-efficient strategy for cardiovascular protection, particularly in populations with limited exercise adherence.

Aerobic exercise may contribute to long-term metabolic homeostasis through its sustained and cumulative effects on myokine secretion. Prolonged moderate-intensity training induces a more persistent IL-6 release pattern [12,13] and effectively modulates FGF21 signaling, enhancing tissue sensitivity and reversing FGF21 resistance. This modality could be considered for establishing baseline metabolic improvements and maintaining cardiovascular health over time.

### 4.2. Myokines as Biomarkers for Exercise Efficacy

The potential of circulating myokines as biomarkers for monitoring exercise efficacy and predicting health outcomes is increasingly recognized, yet several challenges remain. First, detection methods lack standardization across studies, limiting comparability and clinical translation. Second, individual factors such as age, gender, and baseline metabolic status significantly influence both basal myokine levels and exercise-induced responses. For instance, exercise increases apelin expression in obese men but not in women [25], highlighting the need for sex-specific considerations. Third, the phenomenon of myokine resistance—observed for irisin [42,43] and FGF21 [51,52] in obese and diabetic states—complicates interpretation, as elevated circulating levels may reflect target tissue insensitivity rather than enhanced function. Longitudinal studies are needed to establish whether changes in myokine profiles predict long-term metabolic and cardiovascular outcomes, and to determine the clinical utility of myokines as biomarkers outside of research settings.

### 4.3. Targeting Myokine Signaling Pathways for Therapeutic Intervention

The recognition of myokines as key mediators of exercise benefits has sparked interest in developing myokine-based or myokine-targeted therapies. MSTN inhibition represents one of the most advanced approaches, with preclinical studies demonstrating that MSTN knockout or pharmacological inhibition increases muscle mass, reduces adiposity, and improves insulin sensitivity [81]. Clinical trials of MSTN inhibitors for sarcopenia and muscular dystrophies are ongoing, though their cardiovascular effects warrant careful evaluation given MSTN’s direct pro-atherogenic actions [60].

FGF21 analogs have shown promise in clinical trials for non-alcoholic fatty liver disease and type 2 diabetes, mirroring the metabolic benefits of exercise [54]. The observation that exercise enhances FGF21 sensitivity in peripheral tissues [20] suggests that combining pharmacological FGF21 augmentation with exercise training could yield synergistic effects. Similarly, apelin-13 and its analogs are being explored for cardiovascular protection, given their ability to improve lipid metabolism, exert anti-inflammatory effects, and stabilize atherosclerotic plaques [62,63].

However, several considerations temper enthusiasm for direct myokine replacement. The complex, context-dependent actions of myokines—exemplified by the dual roles of IL-6 in inflammation and metabolism—caution against simplistic supplementation approaches. The pulsatile nature of exercise-induced myokine release may be critical for their beneficial effects, a pattern difficult to replicate pharmacologically. Moreover, the interconnected myokine network suggests that targeting single factors may not recapitulate the multi-system benefits of exercise.

### 4.4. Methodological Considerations and Sources of Heterogeneity

Interpretation of myokine responses to exercise is complicated by several confounding variables that are often insufficiently controlled in primary studies. Sex differences may play a role: for example, apelin has been reported to increase after exercise in obese men but not in women [25], and most included studies have predominantly male samples, which may limit generalizability to females. Age may also influence responses; some studies suggest that older adults show blunted irisin responses to training compared with younger populations [40,77].

Training status can affect both baseline and exercise-induced myokine levels. For instance, individuals with metabolic syndrome have been found to exhibit attenuated irisin responses [19], and failure to distinguish between acute and chronic measurement time points may have contributed to some contradictory findings in the literature [18,42,50,80]. Closely related is obesity severity: myostatin levels have been shown to rise with increasing obesity and to correlate with insulin resistance [55], whereas Metrnl levels may progressively decrease as obesity worsens [68].

Other less frequently discussed confounders include medication use (statins, metformin, GLP-1 agonists could influence circulating myokine levels independently of exercise, but most primary studies do not account for this), circadian and nutritional state (many myokines exhibit diurnal rhythms and may be sensitive to fasting/feeding status), and assay variability (irisin measurements, for example, have been reported to vary substantially across different ELISA kits [42]). Despite these sources of heterogeneity, the patterns observed across studies—when interpreted with attention to these factors—provide a coherent framework for understanding exercise-induced myokine regulation in obesity.

## 5. Future Perspective

Despite growing knowledge of exercise-induced myokines, several fundamental gaps remain. A primary obstacle is methodological heterogeneity. Wide variation in ELISA kits, sampling protocols, and circadian timing across studies makes direct comparisons unreliable, and until standardized reference methods are established and validated, circulating myokines will not serve as robust clinical biomarkers. In addition, population-specific reference values—stratified by age, sex, and body mass index—are lacking; large-scale cohort studies are needed to establish these normative ranges. A related conceptual challenge is distinguishing between genuine myokine secretion and compensatory upregulation during tissue resistance; the same circulating level may reflect benefit in one metabolic state but pathology in another. Addressing this requires simultaneous measurement of ligand and receptor signaling—for example, assessing phosphorylation status of downstream effectors in target tissues—rather than relying solely on circulating concentrations.

Another major limitation is that most studies focus on single myokines, yet these molecules operate as an interacting network. Multi-omic profiling of skeletal muscle and blood from the same individuals before and after exercise, combined with pathway analysis, could capture synergistic effects that single-factor approaches miss. Specific mechanistic uncertainties also persist, including the identity of the irisin receptor, the balance between classical and trans-signaling of IL-6 in different vascular beds, and the molecular basis of FGF21 resistance. Resolving these issues will require human biopsy studies, phosphoproteomics, and, where appropriate, tissue-specific genetic models. To translate myokine knowledge into actual therapies, specific steps include: (1) small-molecule screens for myokine receptor agonists; (2) phase I/II trials of recombinant myokines (e.g., apelin-13, FGF21 analogs) in obese patients; and (3) development of intermittent delivery systems to mimic pulsatile release. In parallel with these drug-oriented efforts, a more immediate translational priority is to use myokine profiles to refine exercise prescriptions—for example, selecting resistance training for irisin elevation and MSTN suppression, or HIIT for Metrnl induction.

## 6. Conclusions

Exercise-induced myokines are key molecular messengers mediating the metabolic and cardiovascular benefits of exercise. Resistance training effectively increases irisin and decreases myostatin, promoting muscle mass and fat browning. HIIT induces rapid IL-6 peaks and elevates Metrnl, enhancing anti-inflammatory responses and cardiac function. Aerobic exercise improves FGF21 sensitivity and supports long-term metabolic homeostasis. These modality-specific myokine profiles provide a molecular basis for hypothesis-driven prescription, though the current evidence is derived from heterogeneous studies without quantitative synthesis. Pending validation in future prospective trials, the present evidence suggests that integrating resistance, aerobic, and HIIT training into comprehensive exercise programmes may benefit obese patients. However, current research has limitations. Most studies focus on single myokines rather than their synergistic network, and detection methods lack standardization. A notable limitation is the predominant focus on male participants with obesity; given that apelin and other myokines exhibit sex-dependent responses to exercise, the conclusions may not be fully generalizable to females. Future research should address these gaps.

## Figures and Tables

**Table 1:** Effects of Different Exercise Modalities on Myokine Secretion Profiles.

Myokine	Key Effects of Exercise Modalities
IL-6	HIIT: May induce a rapid and sharp peak [12,13,14]. Aerobic Exercise: May lead to a more sustained and cumulative secretion pattern [14,15]. Resistance Training: Can elevate circulating levels; changes may be less sensitive to intensity variations [12,13].
Irisin	Resistance Training: Likely an efficient mode for its release, potentially superior to aerobic training [16,17]. HIIT: May be an effective strategy for a rapid increase, correlated with exercise intensity [18,19]. Long-term Training: Effects are controversial (increase, decrease, or no change) [17].
FGF21	Resistance & Aerobic Exercise: Both are effective; resistance training may induce a more substantial adaptive response [20].
MSTN	Resistance Training: The most effective way to downregulate its expression, showing a dose-dependent effect [21]. Aerobic Exercise: Most studies report no significant change [22].
Apelin	Inconsistent Findings: Evidence varies across studies on aerobic, HIIT, and resistance training; responses show significant individual variability [23,24,25,26].
Metrnl	HIIT & Resistance Training: Typically elicit more pronounced responses; HIIT may be superior to moderate-intensity continuous training [27,28]. Acute vs. Chronic: Acute exercise increases mRNA, while chronic training elevates circulating protein levels [27,28].

Table created by the authors.

**Table 2:** Exercise prescription framework translating myokine molecular evidence into practical recommendations.

Target Myokine	Exercise Modality	Frequency	Intensity	Duration	Expected Effect	Ref
↑ IL-6 (pulsatile, anti-inflammatory)	HIIT or prolonged moderate aerobic	3 d/wk	85–95% HRpeak (HIIT)/60–70% HRmax (aerobic)	4 × 4 min intervals or 45–60 min continuous	↑ IL-10, ↑ IL-1Ra, systemic anti-inflammation	[14,79]
↑ Irisin	Resistance	3 d/wk	70–80% 1RM	45 min	Browning, ↑ energy expenditure	[16,17,19]
↓ MSTN	Resistance	3–4 d/wk	60–80% 1RM	50 min	↑ muscle mass, ↓ fat	[21]
↑ FGF21 sensitivity	Moderate aerobic	5 d/wk	50–70% HRmax	30–45 min	↑ insulin sensitivity, ↓ steatosis	[80]
↑ Metrnl	HIIT	3 d/wk	85–95% HRpeak	30 min (intervals)	↑ cardiac function, ↓ inflammation	[27]
↑ Apelin	Aerobic/HIIT	3–5 d/wk	60–85% HRmax	30–60 m		[23,24]

Notes: 1RM = one-repetition maximum; HRpeak = peak heart rate; HRmax = maximum heart rate. Prescriptions are preliminary and based on human studies cited; individual adjustments may be needed based on fitness level and comorbidities. Table created by the authors.

## Data Availability

Not applicable.

## Supplementary Materials

The following supporting information can be downloaded at https://www.mdpi.com/article/10.3390/sports14050212/s1: Supplementary File S1: Detailed search strategies for PubMed, Scopus, and Google Scholar; Supplementary Table S1: Characteristics of included studies (first author, year, study design, participant characteristics, exercise modality, myokine assay, timing, and main findings); Supplementary Table S2: SANRA quality assessment checklist with item-by-item scores.

## Abbreviations

AMPK	AMP-activated protein kinase
APJ	Apelin receptor
ASCVD	Atherosclerotic cardiovascular disease
BDNF	Brain-derived neurotrophic factor
BMI	Body mass index
eNOS	Endothelial nitric oxide synthase
ERK	Extracellular signal-regulated kinase
FGF21	Fibroblast growth factor 21
FGFR1	Fibroblast growth factor receptor 1
FNDC5	Fibronectin type III domain-containing protein 5
FOXO3	Forkhead box O3
GDF-8	Growth differentiation factor 8
GLUT4	Glucose transporter type 4
HDL-C	High-density lipoprotein cholesterol
HIIT	High-intensity interval training
HOMA-IR	Homeostatic model assessment for insulin resistance
IL-6	Interleukin-6
IL-10	Interleukin-10
IL-13	Interleukin-13
IL-15	Interleukin-15
IL-1Ra	IL-1 receptor antagonist
JNK	c-Jun N-terminal kinase
LDL	Low-density lipoprotein
LDL-C	Low-density lipoprotein cholesterol
MAPK	Mitogen-activated protein kinase
MCP-1	Monocyte chemoattractant protein-1
MDP	Mitochondrial-derived peptide
Metrnl	Meteorin-like protein
MMP	Matrix metalloproteinase
mTOR	Mammalian target of rapamycin
MSTN	Myostatin
NAFLD	Nonalcoholic fatty liver disease
NF-κB	Nuclear factor kappa-B
NO	Nitric oxide
PCSK9	Proprotein convertase subtilisin/kexin type 9
PGC-1α	Peroxisome proliferator-activated receptor gamma coactivator 1-alpha
PI3K	Phosphoinositide 3-kinase
PPAR	Peroxisome proliferator-activated receptor
RACK1	Receptor for activated C kinase 1
ROS	Reactive oxygen species
SIRT1	Sirtuin 1
SIRT3	Sirtuin 3
SMAD	Mothers against decapentaplegic homolog
TGF-β	Transforming growth factor-beta
TNF-α	Tumor necrosis factor-alpha
UCP1	Uncoupling protein 1
VCAM-1	Vascular cell adhesion molecule-1
