# Supplementary material for: Exercise-Induced Myokines in Obesity-Related Metabolic Disorders and Cardiovascular Protection: A Narrative Review
Source: Sports (Basel). 2026 May 21;14(5):212. doi: 10.3390/sports14050212 (PMC13210986; doi:10.3390/sports14050212)
Supplement: Supplementary file 1 [file sports-14-00212-s001.zip › sports-4243596-Supplementary Material.pdf]

Supplementary Methods. Detailed search strategies

PubMed (searched via https://pubmed.ncbi.nlm.nih.gov/):

("myokines"[MeSH Terms] OR "myokine"[Title/Abstract] OR "irisin"[Title/Abstract] OR "FGF21"[Title/Abstract] OR "myostatin"[Title/Abstract] OR "MSTN"[Title/Abstract] OR "apelin"[Title/Abstract] OR "Metrnl"[Title/Abstract] OR "IL-6"[Title/Abstract]) AND ("obesity"[MeSH Terms] OR "obesity"[Title/Abstract] OR "overweight"[Title/Abstract] OR "adiposity"[Title/Abstract]) AND ("resistance training"[Title/Abstract] OR "aerobic exercise"[Title/Abstract] OR "high-intensity interval training"[Title/Abstract] OR "HIIT"[Title/Abstract])

Scopus (searched via https://www.scopus.com):

(TITLE-ABS-KEY("myokines") OR TITLE-ABS-KEY("irisin") OR TITLE-ABS-KEY("FGF21") OR TITLE-ABS-KEY("myostatin") OR TITLE-ABS-KEY("MSTN") OR TITLE-ABS-KEY("apelin") OR TITLE-ABS-KEY("Metrnl") OR TITLE-ABS-KEY("IL-6")) AND (TITLE-ABS-KEY("obesity") OR TITLE-ABS-KEY("overweight") OR TITLE-ABS-KEY("adiposity")) AND (TITLE-ABS-KEY("exercise") OR TITLE-ABS-KEY("resistance training") OR TITLE-ABS-KEY("aerobic exercise") OR TITLE-ABS-KEY("high-intensity interval training") OR TITLE-ABS-KEY("HIIT"))

Note: Results were manually filtered for English language and human studies after the search, as the Scopus syntax used above does not include direct language/human limits.

Google Scholar (searched via https://scholar.google.com):

Due to Google Scholar's limited advanced search options and frequent changes in its interface, the search string used was:

("myokines" OR "irisin" OR "FGF21" OR "myostatin" OR "MSTN" OR "apelin" OR "Metrnl" OR "IL-6") AND ("obesity" OR "overweight" OR "adiposity") AND ("exercise" OR "resistance training" OR "aerobic exercise" OR "HIIT")

The first 200 results were screened manually for relevance, as Google Scholar does not provide a complete export of all hits.
