# Supplementary material for: Exercise-Induced Myokines in Obesity-Related Metabolic Disorders and Cardiovascular Protection: A Narrative Review
Source: Sports (Basel). 2026 May 21;14(5):212. doi: 10.3390/sports14050212 (PMC13210986; doi:10.3390/sports14050212)
Supplement: Supplementary file 1 [file sports-14-00212-s001.zip › sports-4243596-Table S2.pdf]

Supplementary Table S2. SANRA quality assessment checklist.

Item	Description	Met?	Score (0-2)	Comment
1	Explanation of the review's importance	Yes	2	Clearly stated in Introduction
2	Statement of the review question(s)	Yes	2	Explicitly defined
3	Description of the literature search	Partially	1	Databases and keywords provided; full search strings in Supplementary Methods
4	Referencing of relevant literature	Yes	2	Comprehensive coverage
5	Scientific reasoning	Yes	2	Logical structure with integration of contradictory findings
6	Appropriate presentation of data	Yes	2	Table 1 and Supplementary Table S1
Total			11/12	

SANRA scores: 0=not met, 1=partially met, 2=fully met
